# Supplementary figures and images for: Structure of Klebsiella pneumoniae adenosine monophosphate nucleosidase
Source: PLoS One. 2022 Oct 20;17(10):e0275023. doi: 10.1371/journal.pone.0275023 (PMC9584410; doi:10.1371/journal.pone.0275023)

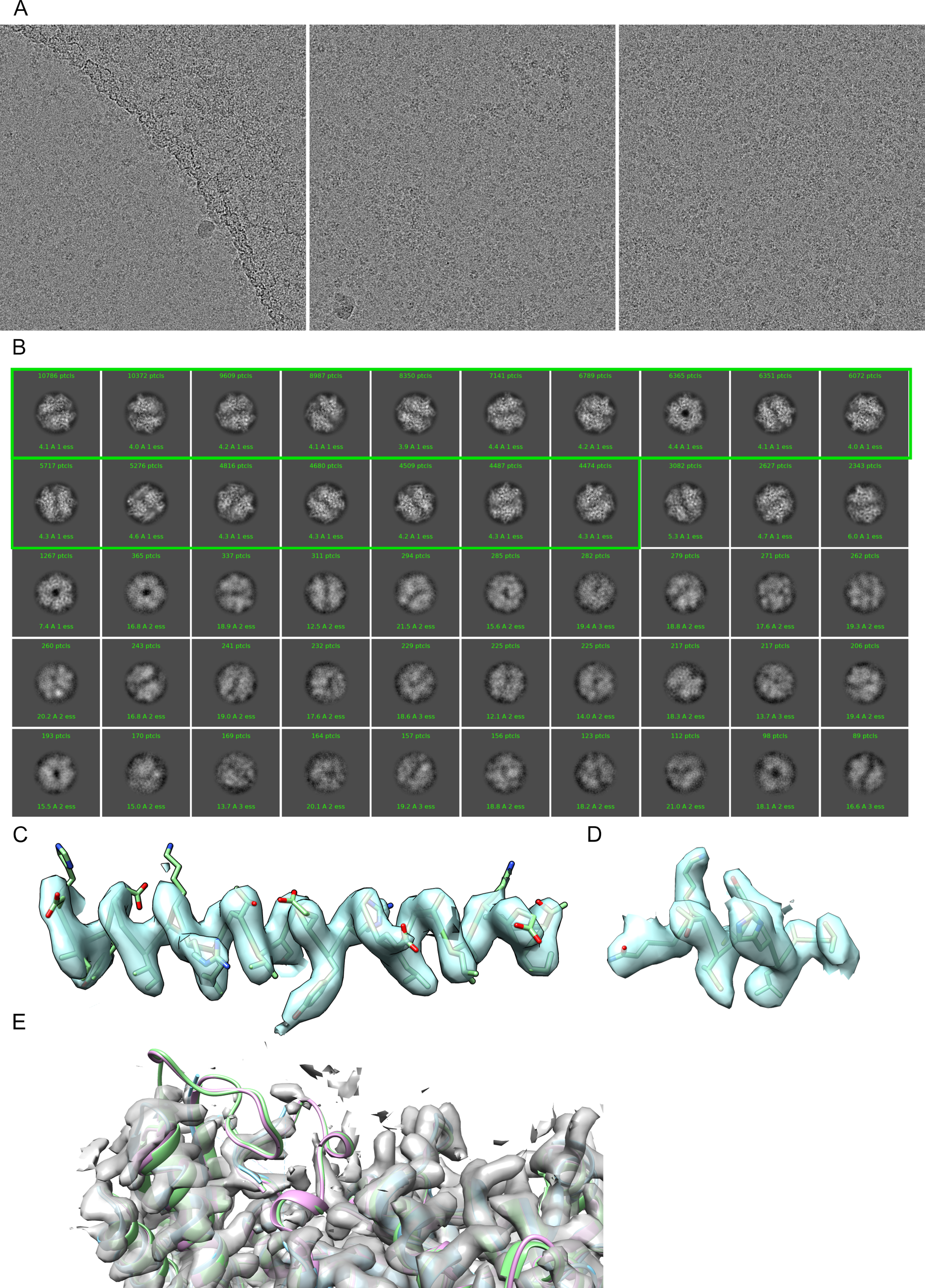

Supplement: S1 Fig — Three randomly selected motion-corrected micrographs with suitable CTF resolution estimates are shown (A). The final density map was obtained from particles in the 2D classes identified by a green border (B). Clear and appropriate density for side chains was evident in both lower (C, residues 8–38) and higher (D, residues 284–294) resolution regions; density is contoured to just above the local noise threshold within 2.4Å of any displayed atom. No KpAmn (blue model) density is observed for the residues corresponding to loops implicated in the E. coli structures’ crystal contacts (green and magenta) (E). (TIF) [file pone.0275023.s001.tif]

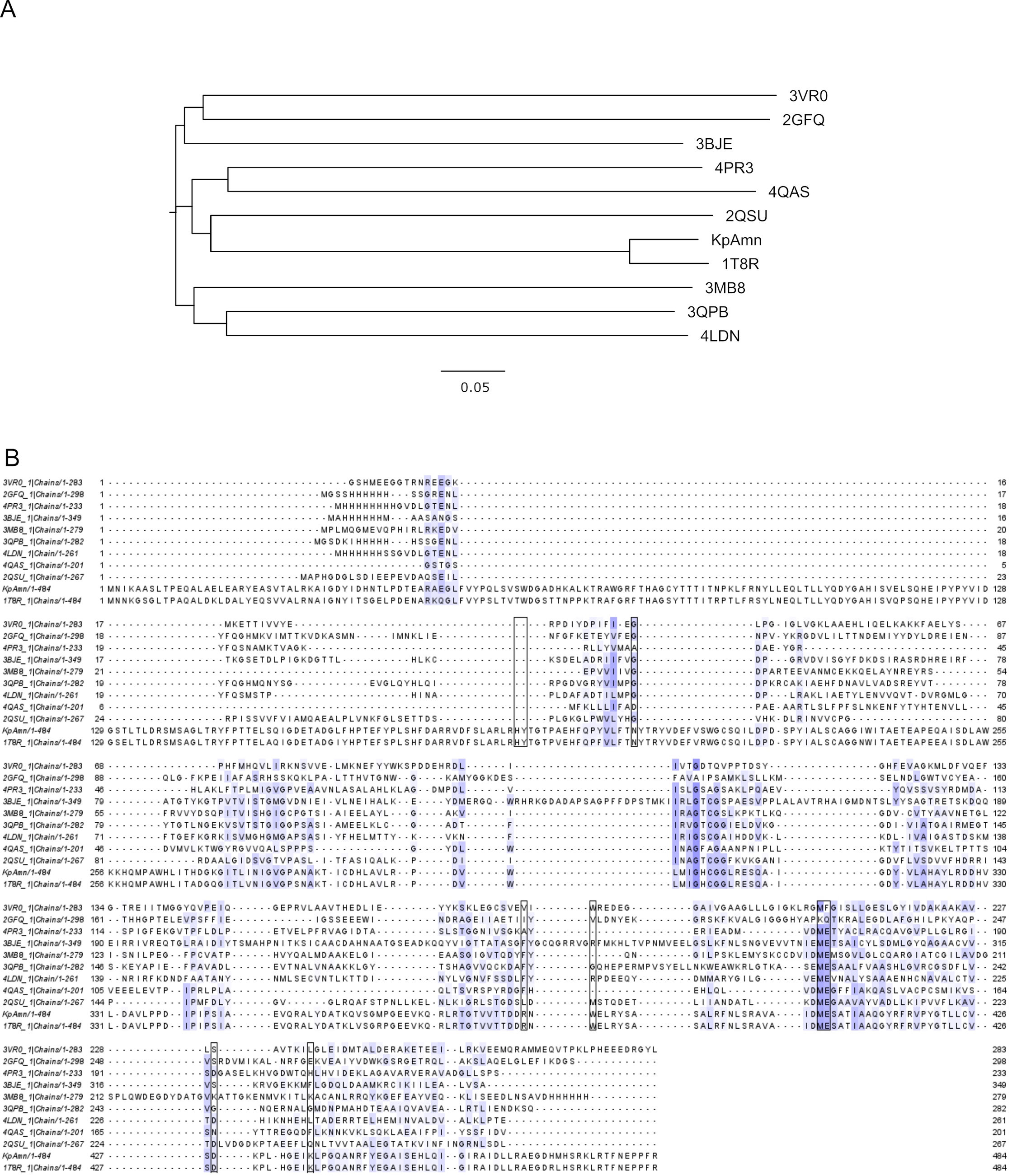

Supplement: S2 Fig — Structural matches to KpAmn identified by DALI were aligned using MUSCLE [37], generating a phylogenetic tree (A) and residue alignment (B). Active site residues are marked with boxes. (TIF) [file pone.0275023.s002.tif]

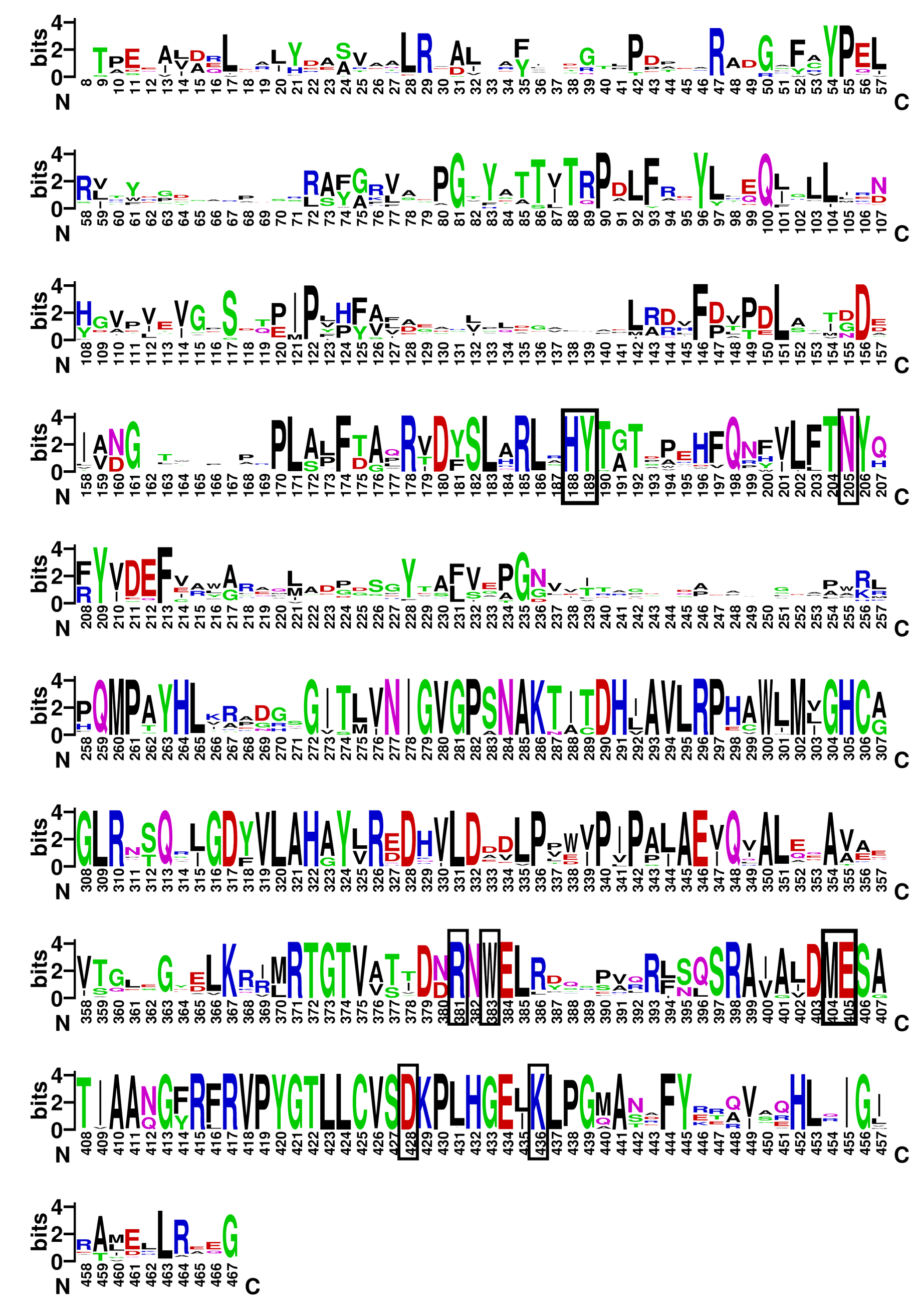

Supplement: S3 Fig — The 150-sequence alignment generated by ConSurf is represented in logo format using WebLogo [38], in which letter height corresponds to strength of individual residue conservation. The N-terminal unresolved residues and C-terminal five residues following a series of unresolved residues are omitted. Active site residues are marked with boxes. (TIF) [file pone.0275023.s003.tif]
