# Supplementary material for: Structure of Klebsiella pneumoniae adenosine monophosphate nucleosidase
Source: PLoS One. 2022 Oct 20;17(10):e0275023. doi: 10.1371/journal.pone.0275023 (PMC9584410; doi:10.1371/journal.pone.0275023)
Supplement: Attachment — Submitted filename: review.docx [file pone.0275023.s004.docx]

Structure of Klebsiella Pneumoniae Adenosine Monophosphate Nucleosidase.

1. The article reports the first cryo-EM structure of an Amn enzyme but no additional structural information is provided by the authors. The authors did not provide any CryoEM micrographs, images, 2D classification images or 3D reconstruction details.
2. As described by authors the EM structure is not providing any new information regarding the KpAmn structure only physiologically irrelevant different loop conformations due crystal contacts in the crystal structure.
3. The authors either rewrite the manuscript in terms of methodology to describe Cryo-EM technique.
