# Supplementary material for: Structure of Klebsiella pneumoniae adenosine monophosphate nucleosidase
Source: PLoS One. 2022 Oct 20;17(10):e0275023. doi: 10.1371/journal.pone.0275023 (PMC9584410; doi:10.1371/journal.pone.0275023)
Supplement: Attachment — Submitted filename: Responses to the reviewer KpAMN 8-4-22.docx [file pone.0275023.s005.docx]

Responses to the reviewer’s comments for PONE-D-22-14060

We thank the reviewers for their insightful comments. We have included all of these comments and a point by point response to each comment below. Our responses are given in blue text and, where possible, include a description of where the modification, edit or addition appears in the revised manuscript.

Reviewer #1: The article reports the first cryo-EM structure of an Amn enzyme except no additional structural information is provided by the authors. The authors did not provide any CryoEM micrographs, images, 2D classification images or 3D reconstruction details.

To address the reviewer’s concerns, we have added a new supplemental figure 1 that includes 3 (truly) randomly selected micrographs (Fig. S1A), the initial 2D classes (including which subset of 2D classes were chosen for further analysis) (Fig. S1B), and several examples of map regions and the resulting models (Figs. S1C-E)

As described by authors the EM structure is not providing any new information regarding the KpAmn structure only physiologically irrelevant different loop conformations due crystal contacts in the crystal structure. The authors either rewrite the manuscript in terms of methodology to describe Cryo-EM technique.

We apologize for the lack of methodological details. As described in further detail below (and in the prior statement), we have included a much more thorough description of processing and refinement.

Reviewer #2: This paper by Richardson et al. describes the cryo- EM structure of adenosine monophosphate nucleosidase (Amn) from a pathogenic bacteria, Klebsiella pneumoniae, which causes hospital-acquired pneumonia and sepsis. Here are the points for revision.

Major points

This manuscript suffers from poor data analysis, the author says in the result, “The cryo-EM structure of K. pneumoniae Amn primarily differs from the apo homologs in its loop regions away from the active site (Figure 4A), either by lacking density for the loop entirely or being in a clearly different conformation”

-In case of lacking density, how the model was built for this region? I do not see any disconnect, missing segment, in Figure 4a.

Figure 4a does not reflect differences clearly, and a magnified view of the loop region would be needed to see the actual conformation differences.

These points have been addressed in supplemental figure 1. Specifically, Figure S1E shows the region in question and illustrates the observed lack of density for the loop region that is well modeled in the corresponding *E. coli* structures.

At 3.1 Å resolution, the difference for side-chain residues should be clear. A picture showing side-chain confirmation would be helpful to reach this conclusion.

These points have been also addressed in supplemental figure 1. Specifically, Figures S1C and S1D show examples of the clear side chain density that our model was built into.

In catalytic activity, the Km and Vmax for the other two species were performed in different experiments, not at the same time and in the same setup. Reaching a conclusion may not be appropriate. Some unnecessary statement “though the --------- intervening decades”, is not required.

We thank the reviewer for this insight and agree with this statement. In light of this, we have deleted the unnecessary statement.

In figure 2B, I don’t see high-resolution features such as density for bulky side chain residues, particularly in a helix. That should be evident at this 3.1 Å resolution, and it looks overall like a low-resolution map. To gain readers’ confidence, detailed cryo-EM data processing information, including representative micrographs, 2D class, 3D class, and final post processed is required.

As described above, we have added further intermediate details about Cryo-EM data analysis in the supplementary Figure 1. This includes sample micrographs, 2D classes, and samples of maps from both high and low-resolution regions of the final maps.

Minor point

In introduction; The acronym ESKAPE in the introduction needs expansion.

We have added the full description of that acronym in the introduction, as suggested.

In result; the Figure 2 parts are not in order e.g. Figure 2C comes first in the text.

The reviewer is correct and we thank them for their thorough analysis. However, we feel that the current layout of Figure 2 is much more logical and aesthetically balanced. As the current way in which the figures are referenced in the text follows the published PLoS ONE guidelines for in-text citations, we respectfully suggest that the organization is both reasonable and within the bounds of PLoS ONE’s formatting and style requirements.

In result; ‘mixed secondary structure’ is not correct, needs to explain in terms of the secondary structure topology or domain. Same in Figure 2 legend also.

We have removed the term “mixed secondary structure” in the description in the results section, and replaced it with the more accurate “extensive β-sheet structure surrounded by α-helices”. In addition, we have removed the word “mixed” from figure 2 caption.

1T8S is a PDB id, it should be written as PDB ID; 1T8S, throughout the text.

We have corrected all instances of this to read “PDB entry XXXX” throughout the text.

In table 1; add one more column for the name of the organism for the source of the protein.

This has been added.

On searching EMD ID 26838 in EMDB shows no entry. Check this out.

The data has been deposited to the PDB and is currently showing that it is *held for publication*.

Reviewer #3: The manuscript describes the structure of K. pneumoniae adenosine monophosphate nucleosidase determined by cryoelectron microscopy and structure is compared with other known structures.

Overall manuscript is informative bur there are several concerns. The overall structure of the enzyme is not described, at least it should have been described briefly.

We have included additional text in the beginning of the results section and in the figure 2 caption briefly describing the overall structure.

There are no images of EM, secondary classification, other than Figure 2B image. At least these should have been in supplementary data.

As described above, we have added further intermediate details about Cryo-EM data analysis in the supplementary Figure 1. This includes sample micrographs, 2D classes, and samples of maps from both high and low-resolution regions of the final maps.

The authors describe structural differences in loops at such a low resolution. Are the lops well defines in EM structure?

The most striking difference is precisely that the loops are less well resolved in the cryo-EM structure, indicative of the lack of crystallographic artifacts. One illustration of this is provided in the new supplementary Figure 1E.

Can they use this low resolution structure for drug design or inhibitor screening?

While the structural information provided could be used as a basis for structure-guided drug development (as described in the discussion section), it is impossible to predict whether specific inhibitors could be resolved in future structures. The quality of the map described herein (illustrated in figures 2, 3 and the new supplementary figure 1) suggests that distinct features of potential inhibitors could be reasonably well resolved, if maps of similar quality were obtained. Having said that, inhibitor bound protein may yield different particle distributions, more or less heterogeneity, and result in substantially better or worse maps.

The manuscript does not describe or give the reference to the literature, explaining the importance of this enzyme. Does the importance of this enzyme shown in this organism?

We have edited the introduction section to emphasize the importance of Amn for the maintenance of nucleotide pools (as essential components of RNA synthesis and energy storage) in this organism, and its value as a drug target. Several appropriate literature references that emphasize these points are included.

Overall the authors should describe the manuscript with more information.

As described above, we have included substantial additional details about the methodology, including additional data describing intermediate processing stages, included sequence alignments (as suggested by reviewer 4) and provided additional details throughout the text as requested by all the reviewers.

Reviewer #4: This paper reports the cryo-EM structure and catalytic activity of Adenosine Monophosphate Nucleosidase from Klebsiella Pneumoniae. However, the mauscript should be revised considering the following comments.

Comment 1: Page3

“Biochemical studies of nucleotide processing pathways in Azotobacter vinelandii and

Escherichia coli (E. coli) identified a key difference between prokaryotes and eukaryotes in their regulation of the levels of adenosine monophosphate (AMP), a key component of both RNA

synthesis and energy storage.” Provide References.

We moved the appropriate reference from the subsequent sentence to be placed immediately following this one, as suggested by the reviewer.

Comment 2: Page 8

All PDB structures identified by BLAST as close Amn homologs were aligned to Amn in UCSF Chimera (A). Provide sequence comparison highlighting the conserved residues and discuss functionally significant residues.

We have added a new figure (supplemental figure 3) that compares and highlights the conservation of all 150 sequences identified by BLAST as close Amn homologs.

Comment 3:

Page 8 line number 145: “broader comparison of K. pneumoniae Amn to its homologs can be assessed by sequence conservation”

Provide the phylogenetic relation between the available structures.

We have added a new figure (supplemental figure 2) that includes the phylogenetic comparison and associated alignment, as suggested by the reviewer.

Comment 4:

Page 8 line number 149: “the loop covering the active site is strongly conserved”

Provide the structural alignment of the active site residues.

An additional panel has been added to figure 3 (Figure 3B), that shows the structural alignment as suggested.

Comment 5:

Page 10 line number 182: “while the crystal structure leads to significant underestimation of the flexibility of several N-terminal loops the catalytic core of the enzyme is effectively invariant between these species.” Discuss any functional significance of the N-terminal loops in Amn, if not then there is no need to include this sentence.

We have removed this sentence.

Comment 6:

The discussion should be precise and elaborate and should only discuss their findings. The KpAmn structure should be compared with that of the other homologs in detail highlighting the functionally relevant substructures.

We have removed, as described above, any extraneous discussion and carefully edited the text to address the comments made by all of the reviewers. We have, in our opinions, included a concise, precise description of our methods, the overall structure, active site, and conserved features of this protein, as well as a brief discussion of the impact. It is unclear what the reviewer means by “precise and elaborate” (which seems to be somewhat contradictory), but we strongly feel that this revised manuscript provides a thorough description and discussion of our findings.
